# Clinical significance of visceral fat reduction through health education in preventing atherosclerotic cardiovascular disease - Lesson from the Amagasaki Visceral Fat Study: A Japanese perspective

**DOI:** 10.1186/1743-7075-8-57

**Published:** 2011-08-16

**Authors:** Ken Kishida, Tohru Funahashi, Iichiro Shimomura

**Affiliations:** 1 Department of Metabolism and Atherosclerosis, Graduate School of Medicine, Osaka University, 2-2 B5 Yamada-oka, Suita, Osaka 565-0871, Japan; 2 Department of Metabolic Medicine, Graduate School of Medicine, Osaka University, 2-2 B5 Yamada-oka, Suita, Osaka 565-0871, Japan

**Keywords:** Visceral fat syndrome, adipocyte dysfunction, health education, Hokenshido, metabolic syndrome

## Abstract

The metabolic syndrome has received worldwide recognition and is useful clinical aid in early-preventing atherosclerosis. Visceral adiposity is the main component of the metabolic syndrome in Japan, based on ethnic and racial difference in the pattern of adiposity. In the Amagasaki Visceral Fat Study, subjects had undergone annual health check-ups and then received health education by medical personnel. Visceral fat reduction improved hypoadiponectinemia and the number of obesity-related cardiovascular risk factors, and effectively prevented cardiovascular events. The health education that includes voluntary lifestyle modification aimed at reducing visceral fat could be useful in preventing cardiovascular events in the metabolic syndrome.

## Introduction

Adipocytes store excess energy as triglyceride, and also function as part of the self-defense system against starvation. Recent advances in research on adipocyte biology have demonstrated that adipocytes synthesize and secrete into the circulation various bioactive substances known as adipocytokines, including angiogenic factors, cytokines, complement factors and anti-diabetic, anti-atherogenic factors, and adiponectin [1]. Masses of adipocytes, i.e., the adipose tissue, exist in many areas of the human body, and play important roles in angiogenesis, wound healing and also act as a mechanical shock absorber. Visceral fat is a specialized adipose tissue located in the mesentery and omentum, and can provide fatty acids and glycerol to the liver directly via the portal vein [2]. However, accumulation of visceral fat exceeding the physiological range results in adipocyte dysfunction. The imbalance in blood levels of adipocytokines; i.e., overproduction of offensive adipocytokines, such as tumor necrosis factor-alpha and plasminogen activator inhibitor-1, and underproduction of defensive adipocytokines such as adiponectin, disturbed metabolism of free fatty acids, and fat-derived reactive oxygen species, is the postulated molecular mechanism of injury of arteries and various organs [2].

### Obesity and lifestyle-related disorders

Obesity is an inevitable physical status in many countries worldwide where physical inactivity and over-eating are common lifestyles, and has become the basis of various lifestyle-related disorders including impaired glucose and lipid metabolism, hypertension, hyperuricemia, chronic kidney disease, sleep apnea-hypopnea syndrome, non-alcoholic steatohepatitis, and atherosclerotic cardiovascular diseases (ACVD). The increased incidence of ACVD in working-age individuals is a worldwide health problem, including East-Asians who have a smaller body mass index compared to their counterparts in Western countries. In Japanese, accumulation of intra-abdominal visceral fat rather than absolute body fat mass seems to be culprit of lifestyle-related disorders including the metabolic syndrome [2]. In this regard, it is important to define the relationship between visceral fat accumulation and lifestyle-related disorders and also the significance of weight reduction, particularly visceral fat reduction, in large-scale general population.

### The Amagasaki Visceral Fat Study

**Figure 1:**
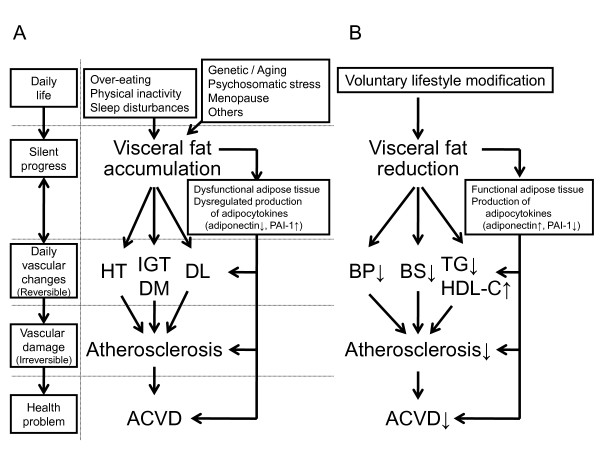
**Lifestyle changes associated with visceral fat and ACVD**. HT, hypertension; IGT, impaired glucose tolerance; DM, diabetes mellitus; DL, dyslipidemia; ACVD, atherosclerotic cardiovascular disease; BP, blood pressure; BS, blood sugar; TG, triglyceride; HDL-C, high density lipoprotein-cholesterol.

In the Amagasaki City Council (a city in Hyogo Prefecture, Japan), several fatal ACVD events, i.e., stroke and myocardial infarction, were recorded between 1994 and 2002 among the ~4,000 employees (Table 1). The insurance and health costs of these events had also increased during the same period. These data call for for programs that can reduce the incidence of fatal ACVD events. In Japan, health education based on the results of the annual health checkups including measurement of visceral adiposity was introduced in 2003 ahead of the theme by the medical staff to prevent lifestyle-related diseases including ACVD. Many people received dietary advice, participated in physical activities and behavior modification, designed to reduce visceral fat and the number of risk factors. Subsequently, evaluation of visceral adiposity, such as measurement of waist circumference, and of visceral fat area, using visceral fat belt [3], was added to the annual medical health check-up. Part of such health education program is run by the public health nurses, who provide the individual with scientific information on the risk factors associated with visceral fat accumulation (silent stage), clustering of multiple risk factors (reversible stage), vascular damage (irreversible stage), and health problems including ACVD events (irreversible and life-threatening stage) (Figure 1A). Furthermore, the same nurses also explained the results of the medical checkup and the relationship between the subject's lifestyle and the abnormalities recorded in the health check-up, to promote voluntary lifestyle changes. Voluntary lifestyle modification resulted in reductions in accumulated visceral fat (reversible stage) (Figure 1B) [4,5]. Another part of the health education program included inviting the subjects to participate in group discussions or one-to-one discussion with the public health nurses. Subjects who required further examination for ACVD or treatment of diabetes, hypertension and/or dyslipidemia, were referred to the family physicians or specialists for further management. Our assessment indicates that this project, called "Hokenshido" in Japanese, has already reduced the incidence of fatal ACVD-related events in urban office workers (Table 1). Health guidance "Hokenshido" is a program that provides explanation to subjects at risk of ACVD about their health condition for disease and motivating them to alter their lifestyle, by public health nurses, dieticians, and physicians when considered necessary. Voluntary lifestyle modification is thus important in the prevention of ACVD.

**Table 1:** Subjects' numbers of fatal atherosclerotic cardiovascular disease events during year 1994-2008

ACVD deaths	1994	1995	1996	1997	1998	1999	2000	2001	2002	2003	2004	2005	2006	2007	2008
Myocardial infarction	2	1	1	2	1	1	2	0	0	0	0	0	1	0	0
Cerebrovascular disease	3	0	0	0	2	0	2	0	2	0	0	0	0	0	1
Total	5	1	1	2	3	1	4	0	2	0	0	0	1	0	1

Numbers of subjects.

A health promotion program directed against the metabolic syndrome was conducted in the Amagasaki municipal government offices, Hyogo prefecture, in 3,946 urban workers from Hyogo. The introduction of this program in year 2003 reduced the number of fatal atherosclerotic cardiovascular disease (ACVD) events (deaths) in that population. Data from Kenko-Amagasakishi 21: Health Survey and Health Promotion Program for staff of the Amagasaki Municipal Government office in 2004.

To understand how such project improved the outcome, we conducted the Amagasaki Visceral Fat Study based on visceral adiposity; a population-based cohort study (UMIN 000002391) [3-12]. It is anticipated that normalization of visceral fat mass through participation in "Hokenshido" resulted in reductions in the incidence of glucose tolerance, dyslipidemia, hypertension [5] and hypoadiponectinemia [7], as well as the number of obesity-related cardiovascular risk factors [4] and hence help effectively prevent ACVD events [11]. Furthermore, by reducing visceral fat mass, "Hokenshido" prevent ACVD events in patients with the metabolic syndrome. Admittedly, our assessment of the health promotion program has certain limitations. First, the study was uncontrolled; i.e., was neither blind nor randomized, and thus, there is a need for a more comprehensive and thorough analysis. Second, we have not all data for non-fatal ACVD events. Finally, in the present study, health checkups were undergone mainly in every May-June. Recent reports have demonstrated that seasonal variations exist within the metabolic syndrome parameters [13-15]. Kamezaki et al. reported that seasonal variations in the metabolic syndrome prevalence in Japanese male workers [15]. The results indicate that the season of health checkups may affect the clinical diagnosis and management of the metabolic syndrome.

## Conclusion

Visceral fat reduction by voluntary lifestyle modification is an important strategy in the prevention of ACVD events in the metabolic syndrome.

## List of abbreviations

ACVD: atherosclerotic cardiovascular diseases

## Competing interests

The authors declare that they have no competing interests.

## Authors' contributions

The authors declare no conflict of interest. KK and TF wrote the manuscript and contributed to the discussion. IS contributed to the discussion. KK reviewed/edited manuscript. All authors read and approved the final version of the manuscript.

## Authors information

Ken Kishida, MD, PhD, is an assistance professor at the Department of Metabolism and Atherosclerosis, Graduate School of Medicine, Osaka University. The focus of Dr. Kishida's research is studying for preventing atherosclerosis based on visceral fat accumulation. Tohru Funahashi, MD, PhD, is a professor in the Department of Metabolism and Atherosclerosis, Graduate School of Medicine, Osaka University. Dr Funahashi's research interests the metabolic syndrome and atherosclerosis. Iichiro Shimomura, MD, PhD, is a professor in the Department of Metabolic Medicine, Graduate School of Medicine, Osaka University. Dr Shimomura's research interests the metabolic syndrome and oxidative stress. Both Dr. Funahashi and Dr. Shimomura have published over 200 peer-reviewed research articles and reviews in international journals.
